# Ultrasonographic findings in mid-trimester adolescent pregnancy: prevalence and risk of abnormalities

**DOI:** 10.3389/fmed.2025.1525149

**Published:** 2025-04-30

**Authors:** Jakub Staniczek, Maisa Manasar-Dyrbuś, Rafał Stojko, Patrycja Sodowska, Magda Rybak-Krzyszkowska, Adrianna Kondracka, Marcin Sadłocha, Krzysztof Sodowski, Agata Włoch, Bartosz Czuba, Wojciech Cnota, Miriam Illa, Agnieszka Drosdzol-Cop

**Affiliations:** ^1^Department of Gynecology, Obstetrics and Gynecological Oncology, Medical University of Silesia, Katowice, Poland; ^2^Department of Gynecology, Obstetrics, Gynecological Oncology, Pediatric and Adolescent Gynecology, Bonifraters’ Medical Center, Katowice, Poland; ^3^Sodowscy Medical Center, Katowice, Poland; ^4^Department of Obstetrics and Perinatology, University Hospital, Krakow, Poland; ^5^Department of Obstetrics and Pathology of Pregnancy, Medical University of Lublin, Lublin, Poland; ^6^Department of Gynecology and Obstetrics, Medical University of Silesia, Ruda Śląska, Poland; ^7^BCNatal Fetal Medicine Research Center, Hospital Clínic and Hospital Sant Joan de Déu, Universitat de Barcelona, Barcelona, Spain

**Keywords:** adolescent pregnancy, teenage pregnancy, fetal abnormalities, maternal-fetal medicine, ultrasonography

## Abstract

**Background:**

Adolescent pregnancy, defined as pregnancy in females aged 19 or younger, is associated with higher risks for adverse outcomes compared to older women. Ultrasound imaging during the mid-trimester is crucial for prenatal care, providing insights into fetal development and maternal health.

**Objective:**

The primary aim of this study is to evaluate and compare mid-trimester ultrasound findings between adolescent and older pregnant women and to assess the prevalence and risk of any abnormalities detected during ultrasound examinations.

**Methods:**

This retrospective study was conducted in the Silesian Voivodeship, Poland, from January 1, 2004, to February 29, 2024. Data were collected from reference prenatal centers. The study included 37,366 individuals, of which 1,152 were adolescents. Participants underwent second-trimester prenatal screening, and ultrasound findings were categorized into fetal abnormalities and maternal factors.

**Results:**

The study found significant differences in ultrasonographic findings across different age groups. Adolescent pregnancies showed a higher prevalence of fetal abnormalities, 437.075 per 1,000 pregnancies (95% CI: 409–465). Brain, spine, facial, heart, and urinary tract abnormalities were notably higher in group adolescents.

**Conclusion:**

Adolescent pregnancies are associated with increased detection of various fetal abnormalities during mid-trimester ultrasound scans. Contrary to common belief, the young age of adolescent pregnant individuals does not protect against fetal abnormalities. These findings underscore the necessity for comprehensive, population-based ultrasound screening for pregnant adolescents and the classification and management of adolescent pregnancies as high-risk.

## 1 Introduction

### 1.1 Background

Adolescent pregnancy, defined by the World Health Organization (WHO) as pregnancy in females aged 19 years or younger, presents significant public health challenges globally. Studies have demonstrated that pregnancies in younger women are associated with a higher risk of congenital anomalies compared to those in older women (1–5).

Loane et al. (3) reported a higher crude prevalence of non-chromosomal anomalies (NCA) among teenage mothers (<20 years) at 26.5 per 1,000 births, compared to lower rates in older age groups. The adjusted relative risk (RR) for teenage mothers was 1.11 (95% CI 1.06–1.17), indicating a significantly higher risk of specific anomalies like gastroschisis and nervous system defects. Chen et al. (4) also found an increased risk of central nervous, gastrointestinal, and musculoskeletal anomalies in adolescent pregnancies. Valim dos Reis et al. (6) and Berham et al. (7) confirmed higher prevalence rates of neural tube and central nervous system defects in adolescent mothers.

The increased risks associated with adolescent pregnancies can be attributed to several factors, including biological immaturity, delayed recognition of pregnancy, socioeconomic disadvantages, substance use during pregnancy, and limited access to healthcare services. These factors often result in inadequate prenatal care, lower awareness of folic acid supplementation, and higher rates of adverse fetal outcomes (1, 7–9).

These findings highlight the importance of targeted interventions to improve prenatal care and education among adolescent mothers to reduce the incidence of congenital anomalies and other adverse outcomes associated with teenage pregnancies. Ultrasound imaging during the mid-trimester of pregnancy is a critical component of prenatal care, offering essential insights into fetal development and maternal health. This publication is among the first to address the prevalence and risk of abnormalities in mid-trimester scans in adolescent pregnancies. In 2023, the authors (1) developed the first Polish standards for managing adolescent pregnancies, emphasizing that this issue is a social and medical challenge and drawing attention to inequalities in access to health services for children. Moreover, studies that provide novel insights into health disparities or inequities, such as those related to adolescent pregnancy, play a key role in advancing health equity. This research also adds significant value to broader public health efforts to improve prenatal care and children and adolescents’ overall well-being and education.

### 1.2 Objectives

The primary objective of this study is to evaluate and compare mid-trimester ultrasound findings between adolescent and older pregnant women and to assess the prevalence and risk of any abnormalities detected during ultrasound examinations.

## 2 Methodology

### 2.1 Study design

This is a retrospective study aimed at examining ultrasound findings in mid-trimester adolescent pregnancies and comparing these results with those from pregnancies of older women. The study was approved by the Review Board of the Chair and Department of Gynecology, Obstetrics, and Gynecological Oncology at the Medical University of Silesia, Katowice. The Bioethics Committee of the Medical University of Silesia, Katowice, Poland (Decision No. BNW/NWN/0052/KB/28/25, dated February 5, 2025) waived the need for ethics approval and the need to obtain consent for the collection, analysis and publication of the retrospectively obtained and anonymized data for this non-interventional study. The study was conducted in accordance with ethical principles governing medical research, including the Declaration of Helsinki. All personal data were securely protected and remained confidential within the participating research center. The paper’s structured methodology adheres to the Strengthening the Reporting of Observational Studies in Epidemiology (STROBE) guidelines to ensure a rigorous and replicable research framework (10).

### 2.2 Settings

The range of data analyzed covered the period from January 1, 2004, to February 29, 2024. During this time frame, all patients who participated in second-trimester prenatal screenings were included in the study, including those who had the examination privately and those covered by the National Program for subsidized prenatal screening. The study was conducted in the Silesian Voivodeship in Poland using data from reference prenatal centers: the Sodowski Medical Center in Katowice, the GENOM (Godula Hope) Medical Center in Ruda Śląska, and the Department of Gynecology, Obstetrics, Gynecological Oncology, Pediatric and Adolescent Gynecology in Katowice. The Chair and Department of Gynecology, Obstetrics, and Oncological Gynecology staff analyzed the collected data at the Medical University of Silesia in Katowice.

### 2.3 Participants

Participants in this study underwent prenatal screening during the mid-trimester of pregnancy (18–25 weeks). The cohort was divided into two main groups: adolescent pregnancies, defined as women 19 years of age or younger, and a control group comprising all other pregnant women. Subsequently, the study population was stratified into three age categories: Group I (19 years and younger), Group II (20–39 years), and Group III (40–47 years).

These specific age brackets (14–19, 20–39, and 40–47 years) were chosen to capture distinct clinical and biological considerations across different stages of reproductive life. Although 35 years is traditionally used to define advanced maternal age, our approach aimed to emphasize both extremes of maternal age—adolescents (14–19 years) and advanced maternal age (40–47 years)—while encompassing the majority of pregnancies in the 20–39-year group. This stratification allows for a more nuanced assessment of the risks associated with both ends of the maternal age spectrum.

### 2.4 Variables

The variables were grouped into categories concerning fetal abnormalities and maternal characteristics. The variables are included in Table 1.

**Table 1:** Grouped variables categories concerning fetal abnormalities and maternal characteristics.

Fetal variables	Maternal variables
Major and minor anatomical abnormalities	Gravidity
Amniotic fluid volume	Ethnicity
Placental abnormalities	Multiple pregnancy
Estimated fetal weight during the scan	Cervical length
Fetal sex	Week of second trimester presentation
Need for invasive testing	Ultrasound in the first trimester
Abnormal karyotype percentages	Smoking status

### 2.5 Data sources

The dataset consists of transabdominal ultrasonographic results recorded during routine prenatal examinations. The data were stored using the Astraia software package from NEXUS / ASTRAIA GmbH. All patient data were anonymized and processed with the following regulations. Additionally, two researchers (M.M-D. and J.S.) independently analyzed and extracted anonymized ultrasound data from the Astraia software.

### 2.6 Bias

Several potential sources of bias were identified, along with strategies specifically designed to mitigate their impact on our findings. All examinations were conducted by certified physicians specializing in Obstetrics and Gynecology, Pediatrics, or Perinatology, who held current prenatal diagnostic certifications issued by the Polish Society of Gynecologists and Obstetricians.

The examinations were performed in accordance with the protocol defined by the Polish Society of Gynecologists and Obstetricians (11, 12) and the ISUOG Practice Guidelines (13–17), using state-of-the-art ultrasound machines, including the GE Voluson Systems (General Electric) and Hera Systems (Samsung). The data from the examinations were recorded using the Astraia software. This approach ensures high quality, consistency, and reliability in the ultrasound examinations, effectively counteracting bias in measuring and recording ultrasonographic findings across all participating centers.

To reduce selection bias, we included a comprehensive cohort of all pregnancies undergoing mid-trimester ultrasonographic examination in the Silesian Voivodeship in Poland over the past twenty years. Missing data can introduce bias and affect the validity of study results, so incomplete patient records were excluded from the study to minimize this risk.

### 2.7 Study size

The sample size comprised a total number of 37,366 individuals, of which 1,152 were adolescent pregnant. The study included 1,332 cases of multiple pregnancies. The total number of fetuses evaluated in the study was 38,737, with 1,176 fetuses from teenage pregnancies.

### 2.8 Statistics methods

All statistical analyses were conducted using the R language in the RStudio environment. The chi-square test was applied to compare qualitative variables across three groups of patients. An analysis of variance (ANOVA) was used for continuous variables to compare mean values between groups. Prevalence (P) was expressed as the number of cases per 1,000 pregnancies, and confidence intervals (95% CI) were calculated to assess the precision of estimations. Comparisons between groups were made using the proportion test, with *p*-values also calculated for pairwise comparisons (Group I vs. Group II, Group I vs. Group III). Using logistic regression models, risk ratios (RR) and 95% confidence intervals (95% CI) were computed to estimate the risk of fetal abnormalities in teenagers compared to the reference group of women aged 20–39. All statistical tests were two-sided, and the level of statistical significance was set at *p* < 0.05.

## 3 Results

### 3.1 Participant’s characteristics

Tables 2, 3 present the study groups’ general characteristics. Appendix 1 provides detailed characteristics of the participants.

**Table 2:** The general characteristics of the study groups.

Variable		GROUP I		GROUP II		GROUP III		*p*-value
		14–19 years		20–39 years		40–47 years		
		*N*=1152		*N*=34192		*N*=2022		
		n/M	%/SD	n/M	%/SD	n/M	%/SD	
Ethnicity	White	1145	99.4%	33991	99.4%	1915	94.7%	>0.001
	Other	6	0.5%	200	0.6%	26	1.3%	
Gravidity	1	1123	97.5%	20618	60.3%	886	43.8%	>0.001
	>1	29	2.5%	13574	39.7%	1140	56.4%	
Tobacco		5	0.4%	208	0.6%	11	0.5%	>0.001
I trimester USG		335	29.1%	27794	81.3%	1714	84.8%	>0.001
Multiple pregnancy		23	2.0%	1262	3.7%	47	2.3%	>0.001
Fetal sex	Girls	588	50.0%	17746	50.0%	1043	50.4%	>0.001
	Boys	581	49.4%	17735	50.0%	1022	49.4%	
	Undiagnosed	7	0.6%	10	0.03%	5	0.2%	
Invasive procedures		102	8.9%	5039	14.7%	751	37.1%	>0.001
Amniocentesis		90	88.2%	4374	86.8%	687	91.5%	0.002
Chorionic Villus Sampling		12	11.8%	665	15.2%	64	8.5%	
Abnormal karyotype		12	1.02%^a^	755	2.2%^a^	122	5.8%^a^	>0.001
			11.8%^b^		15,0%^b^		16.3%^b^	0.426

a % to all fetuses.

b % to invasive procedures.

**Table 3:** The general characteristics of the study groups.

Variable		GROUP I	GROUP II	GROUP III
		14–19 years	20–39 years	40–47 years
Gestational age	Weeks	22	20	20
	Days	5	2	2
Fetuses (*n*)		1176	35491	2070
Estimated fetal weight (g)		433	360	362

The study included participants categorized into three age groups: Group I (14–19 years, *N* = 1,152), Group II (20–39 years, *N* = 34,192), and Group III (40–47 years, *N* = 2,022). The total number of fetuses was 1,176 for Group I, 35,491 for Group II, and 2,070 for Group III.

The mean gestational age (GA) during mid-trimester scan was higher in Group I by over 2 weeks, averaging 22 weeks compared to 20 weeks for both Groups II and III, with mean days of 5 for Group I and 2 for Groups II and III. The ethnicity distribution showed a predominance of White participants in all groups (Group I: 99.4%, Group II: 99.4%, Group III: 94.7%) with statistical significance (*p* > 0.001).

Gravidity was significantly different among groups, with most participants in Group I having a gravidity of 1 (97.48%), compared to 60.30% in Group II and 43.82% in Group III (*p* > 0.001). Tobacco use was reported at 0.43% in Group I, 0.61% in Group II, and 0.54% in Group III (*p* > 0.001). First-trimester ultrasound (US) utilization was lower in Group I (29.08%) compared to Groups II and III (81.29 and 84.77%, respectively, *p* > 0.001).

The incidence of multiple pregnancies was 2.00% in Group I, 3.69% in Group II, and 2.32% in Group III (*p* > 0.001). The estimated fetal weight (EFW) at the time of the study was higher in Group I, with a mean of 433 grams, compared to 360 grams in Group II and 362 grams in Group III. Fetal sex distribution showed a balanced ratio of girls and boys across all groups, with significant *p*-values (*p* > 0.001).

Invasive procedures were less common in Group I (8.85%) compared to Group II (14.74%) and Group III (37.14%), with amniocentesis being the predominant procedure in all groups (Group I: 88.2%, Group II: 86.80%, Group III: 91.5%, *p* = 0.002). The occurrence of abnormal karyotypes increased with age (Group I: 1.02%, Group II: 2.2%, Group III: 5.9%, *p* > 0.001).

### 3.2 Prevalence for fetal abnormalities

Group I exhibited the highest prevalence of fetal abnormalities identified during mid-trimester anatomy scans, with a rate of 437.075 per 1,000 pregnancies (95% CI: 409–465). This was significantly higher compared to Group III, which had a prevalence of 158.937 per 1,000 pregnancies (95% CI: 143–175), and Group II, which showed the lowest prevalence at 139.472 per 1,000 pregnancies (95% CI: 136–143) (*p* < 0.001 for Group I vs. II and Group I vs. III). The prevalence of fetal anomalies in the three groups is presented in Table 4.

**Table 4:** The prevalence of fetal abnormalities in the study groups.

Fetal abnormalities (by system and condition)	GROUP I				GROUP II				GROUP III				*p*-value	*p*-value	*p*-value
	14–19 years				20–39 years				40–47 years					I vs II	I vs III
	*N*	P	95% CI		*N*	P	95% CI		*N*	P	95% CI				
All abnormalities	514	437.075	409	465	4950	139.472	136	143	329	158.937	143	175	<0.001	<0.001	<0.001
Brain	88	74.830	60	90	487	13.722	13	15	31	14.976	10	20	<0.001	<0.001	<0.001
Cerebellar hypoplasia	5	4.252	0.5	8	5	0.141	0	0.3	0	0.000	0	0	<0.001	<0.001	0.012
Choroid plexus cysts	27	22.959	14.4	31.5	349	9.833	8.8	10.9	18	8.696	4.7	12.7	<0.001	<0.001	0.001
Blake’s pouch cyst	2	1.701	0	4.1	2	0.056	0	0.1	0	0.000	0	0	<0.001	<0.001	0.254
Holoprosencephaly	10	8.503	3.3	13.8	1	0.028	0	0.1	0	0.000	0	0	<0.001	<0.001	<0.001
Megacisterna magna	4	3.401	0.1	6.7	5	0.141	0	0.3	0	0.000	0	0	<0.001	<0.001	0.033
Corpus callosum agenesis	7	5.952	1.6	10.3	8	0.225	0.1	0.4	0	0.000	0	0	<0.001	<0.001	0.002
Dolichocephaly	5	4.252	0.5	8	18	0.507	0.3	0.7	0	0.000	0	0	<0.001	<0.001	0.012
Brachycephaly	1	0.850	0	2.5	46	1.296	0.9	1.7	10	4.831	1.8	7.8	<0.001	0.995	0.118
Microcephaly	6	5.102	1	9.2	11	0.310	0.1	0.5	0	0.000	0	0	<0.001	<0.001	0.005
Craniosynostosis	1	0.850	0	2.5	3	0.085	0	0.2	0	0.000	0	0	0.035	0.292	0.775
Acranius	4	3.401	0.1	6.7	18	0.507	0.3	0.7	0	0.000	0	0	<0.001	0.001	0.033
Ventriculomegaly	14	11.905	5.7	18.1	8	0.225	0.1	0.4	2	0.966	0	2.3	<0.001	<0.001	<0.001
Dandy-Walker Syndrome	2	1.701	0	4.1	13	0.366	0.2	0.6	1	0.483	0	1.4	0.085	0.135	0.620
Spine	10	8.503	3.3	13.8	105	2.958	2.4	3.5	3	1.449	0	3.1	0.001	0.002	0.006
Open spina bifida	10	8.503	3.3	13.8	105	2.958	2.4	3.5	3	1.449	0	3.1	0.001	0.002	0.006
Face	27	22.959	14.4	31.5	302	8.509	7.6	9.5	28	13.527	8.6	18.5	<0.001	<0.001	0.063
Cleft lip	9	7.653	2.7	12.6	22	0.620	0.4	0.9	1	0.483	0	1.4	<0.001	<0.001	0.001
Cleft lip and palate	6	5.102	1	9.2	85	2.395	1.9	2.9	3	1.449	0	3.1	0.116	0.124	0.120
Micrognathia	5	4.252	0.5	8	72	2.029	1.6	2.5	5	2.415	0.3	4.5	0.252	0.189	0.563
Nasal bone hypoplasia	3	2.551	0	5.4	79	2.226	1.7	2.7	19	9.179	5.1	13.3	<0.001	1.000	0.047
Hyportelorism	2	1.701	0	4.1	25	0.704	0.4	1	0	0.000	0	0	0.207	0.488	0.254
Hypertelorism	2	1.701	0	4.1	19	0.535	0.3	0.8	0	0.000	0	0	0.133	0.306	0.254
Neck	4	3.401	0.1	6.7	73	2.057	1.6	2.5	7	3.382	0.9	5.9	0.296	0.505	1.000
Cystic hygroma	1	0.850	0	2.5	33	0.930	0.6	1.2	0	0.000	0	0	0.381	1.000	0.775
Nuchal edema	3	2.551	0	5.4	37	1.043	0.7	1.4	7	3.382	0.9	5.9	0.005	0.275	0.935
Cervical teratoma	0	0.000	0	0	3	0.085	0	0.2	0	0.000	0	0	0.872	1.000	NaN
Cervical lymphangioma	0	0.000	0	0	5	0.141	0	0.3	0	0.000	0	0	0.796	1.000	NaN
Thorax	11	9.354	3.9	14.9	154	4.339	3.7	5	7	3.382	0.9	5.9	0.030	0.021	0.050
Diaphragmatic hernia	6	5.102	1	9.2	72	2.029	1.6	2.5	3	1.449	0	3.1	0.061	0.054	0.120
Congenital pulmonary airway malformation	3	2.551	0	5.4	28	0.789	0.5	1.1	0	0.000	0	0	0.046	0.125	0.090
Lung agenesis	1	0.850	0	2.5	9	0.254	0.1	0.4	2	0.966	0	2.3	0.114	0.748	1.000
Pleural effusion	1	0.850	0	2.5	37	1.043	0.7	1.4	2	0.966	0	2.3	0.975	1.000	1.000
Pulmonary sequestration	0	0.000	0	0	8	0.225	0.1	0.4	0	0.000	0	0	0.694	1.000	NaN
Heart and vessels	230	195.578	172.9	218.2	2528	71.229	68.6	73.9	186	89.855	77.5	102.2	<0.001	<0.001	<0.001
Ventricular Septal Defect (VSD)	23	19.558	11.6	27.5	296	8.340	7.4	9.3	56	27.053	20.1	34	<0.001	<0.001	0.225
Atrioventricular Septal Defect (AVSD)	2	1.701	0	4.1	68	1.916	1.5	2.4	9	4.348	1.5	7.2	0.056	1.000	0.351
Hyperechogenic focus	97	82.483	66.8	98.2	1516	42.715	40.6	44.8	85	41.063	32.5	49.6	<0.001	<0.001	<0.001
Tricuspid regurgitation	14	11.905	5.7	18.1	148	4.170	3.5	4.8	7	3.382	0.9	5.9	<0.001	<0.001	0.007
Mitral regurgitation	0	0.000	0	0	11	0.310	0.1	0.5	0	0.000	0	0	0.605	1.000	NaN
Cardiomegaly	3	2.551	0	5.4	30	0.845	0.5	1.1	2	0.966	0	2.3	0.159	0.154	0.521
Supraventricular arrhythmia	25	21.259	13	29.5	47	1.324	0.9	1.7	3	1.449	0	3.1	<0.001	<0.001	<0.001
Atrioventricular block	1	0.850	0	2.5	3	0.085	0	0.2	0	0.000	0	0	0.035	0.292	0.775
Tachycardia	10	8.503	3.3	13.8	26	0.733	0.5	1	1	0.483	0	1.4	<0.001	<0.001	0.001
Interrupted Aortic Arch (IAA)	2	1.701	0	4.1	5	0.141	0	0.3	0	0.000	0	0	<0.001	0.006	0.254
Aortic coarctation	3	2.551	0	5.4	15	0.423	0.2	0.6	1	0.483	0	1.4	0.005	0.010	0.274
Persistent Right Umbilical Vein (PRUV)	4	3.401	0.1	6.7	0	0.000	0	0	1	0.483	0	1.4	<0.001	<0.001	0.116
Persistent Left Superior Vena Cava (PLSVC)	2	1.701	0	4.1	0	0.000	0	0	0	0.000	0	0	<0.001	<0.001	0.254
Transposition of the Great Arteries (TGA)	7	5.952	1.6	10.3	47	1.324	0.9	1.7	2	0.966	0	2.3	<0.001	<0.001	0.025
Tetralogy of Fallot (ToF)	3	2.551	0	5.4	44	1.240	0.9	1.6	1	0.483	0	1.4	0.274	0.411	0.274
Double outlet right ventricle	2	1.701	0	4.1	23	0.648	0.4	0.9	1	0.483	0	1.4	0.369	0.428	0.620
Double inlet left ventricle (DILV)	0	0.000	0	0	4	0.113	0	0.2	0	0.000	0	0	0.833	1.000	NaN
Hypoplastic left heart syndrome (HLHS)	5	4.252	0.5	8	51	1.437	1	1.8	0	0.000	0	0	0.009	0.040	0.012
Hypoplastic Right Heart Syndrome (HRHS)	1	0.850	0	2.5	4	0.113	0	0.2	0	0.000	0	0	0.079	0.389	0.775
Dextrocardia	2	1.701	0	4.1	18	0.507	0.3	0.7	0	0.000	0	0	0.118	0.276	0.254
Hypertrophic cardiomyopathy	2	1.701	0	4.1	9	0.254	0.1	0.4	1	0.483	0	1.4	0.019	0.050	0.620
Heterotaxy	1	0.850	0	2.5	9	0.254	0.1	0.4	0	0.000	0	0	0.344	0.748	0.775
Agenesis of Ductus Venosus	5	4.252	0.5	8	3	0.085	0	0.2	0	0.000	0	0	<0.001	<0.001	0.012
Agenesis of Inferior Vena Cava	2	1.701	0	4.1	9	0.254	0.1	0.4	0	0.000	0	0	0.011	0.050	0.254
Right Aortic Arch (RAA)	2	1.701	0	4.1	10	0.282	0.1	0.5	1	0.483	0	1.4	0.031	0.068	0.620
Pulmonary Atresia (PA)	1	0.850	0	2.5	14	0.394	0.2	0.6	1	0.483	0	1.4	0.741	0.978	1.000
Tricuspid Valve Atresia	1	0.850	0	2.5	14	0.394	0.2	0.6	1	0.483	0	1.4	0.741	0.978	1.000
Aortic Valve Atresia	6	5.102	1	9.2	14	0.394	0.2	0.6	1	0.483	0	1.4	<0.001	<0.001	0.020
Aberrant right subclavian artery (ARSA)	4	3.401	0.1	6.7	17	0.479	0.3	0.7	0	0.000	0	0	<0.001	0.001	0.033
Persistent left superior vena cava (PLSVC)	0	0.000	0	0	15	0.423	0.2	0.6	4	1.932	0	3.8	0.008	1.000	0.323
Tumors	0	0.000	0	0	6	0.169	0	0.3	4	1.932	0	3.8	<0.001	1.000	0.323
Truncus Arteriosus Communis (TAC)	0	0.000	0	0	11	0.310	0.1	0.5	0	0.000	0	0	0.605	1.000	NaN
Ebstein's anomaly	0	0.000	0	0	9	0.254	0.1	0.4	1	0.483	0	1.4	0.700	1.000	1.000
Hypoplastic aortic arch	0	0.000	0	0	9	0.254	0.1	0.4	0	0.000	0	0	0.663	1.000	NaN
Single Ventricle Defect	0	0.000	0	0	9	0.254	0.1	0.4	3	1.449	0	3.1	0.009	1.000	0.481
Hart ectopy	0	0.000	0	0	1	0.028	0	0.1	0	0.000	0	0	0.955	1.000	NaN
Situs inversus	0	0.000	0	0	13	0.366	0.2	0.6	0	0.000	0	0	0.552	1.000	NaN
Abdominal wall	38	32.313	22.2	42.4	119	3.353	2.8	4	5	2.415	0.3	4.5	<0.001	<0.001	<0.001
Gastroschisis	31	26.361	17.2	35.5	41	1.155	0.8	1.5	0	0.000	0	0	<0.001	<0.001	<0.001
Omphalocele	7	5.952	1.6	10.3	72	2.029	1.6	2.5	5	2.415	0.3	4.5	0.017	0.011	0.195
Body-Stalk Anomaly	0	0.000	0	0	6	0.169	0	0.3	0	0.000	0	0	0.760	1.000	NaN
Gastrointestinal tract	9	7.653	2.7	12.6	322	9.073	8.1	10.1	24	11.594	7	16.2	0.433	0.727	0.371
Esophageal atresia	7	5.952	1.6	10.3	6	0.169	0	0.3	0	0.000	0	0	<0.001	<0.001	0.002
Duodenal atresia	0	0.000	0	0	19	0.535	0.3	0.8	3	1.449	0	3.1	0.168	0.887	0.481
Small bowel obstruction	2	1.701	0	4.1	9	0.254	0.1	0.4	0	0.000	0	0	0.011	0.050	0.254
Hyperechogenic bowel	0	0.000	0	0	250	7.044	6.2	7.9	17	8.213	4.3	12.1	0.012	0.007	0.004
Ascites	0	0.000	0	0	37	1.043	0.7	1.4	4	1.932	0	3.8	0.253	0.522	0.323
Liver Tumors	0	0.000	0	0	1	0.028	0	0.1	0	0.000	0	0	0.955	1.000	NaN
Urinary tract	62	52.721	39.9	65.5	576	16.229	14.9	17.5	27	13.043	8.2	17.9	<0.001	<0.001	<0.001
Megacistis	3	2.551	0	5.4	8	0.225	0.1	0.4	0	0.000	0	0	<0.001	<0.001	0.090
Autosomal dominant polycystic kidneys	6	5.102	1	9.2	0	0.000	0	0	0	0.000	0	0	<0.001	<0.001	0.005
Autosomal recessive polycystic kidneys	1	0.850	0	2.5	0	0.000	0	0	0	0.000	0	0	<0.001	0.008	0.775
Polycystic kidneys	0	0.000	0	0	44	1.240	0.9	1.6	4	1.932	0	3.8	0.323	0.435	0.323
Multicystic kidneys	0	0.000	0	0	65	1.831	1.4	2.3	0	0.000	0	0	0.051	0.264	NaN
Hydronephrosis	38	32.313	22.2	42.4	348	9.805	8.8	10.8	18	8.696	4.7	12.7	<0.001	<0.001	<0.001
Pyelectasis	8	6.803	2.1	11.5	46	1.296	0.9	1.7	5	2.415	0.3	4.5	<0.001	<0.001	0.107
Kidney agenesis	4	3.401	0.1	6.7	57	1.606	1.2	2	0	0.000	0	0	0.056	0.262	0.033
Kidney dysplasia	0	0.000	0	0	7	0.197	0.1	0.3	0	0.000	0	0	0.726	1.000	NaN
Bladder exstrophy	2	1.701	0	4.1	1	0.028	0	0.1	0	0.000	0	0	<0.001	<0.001	0.254
Genital tract	6	5.102	1	9.2	15	0.423	0.2	0.6	0	0.000	0	0	<0.001	<0.001	0.005
Ovarian cyst	5	4.252	0.5	8	13	0.366	0.2	0.6	0	0.000	0	0	<0.001	<0.001	0.012
Hypospadias	1	0.850	0	2.5	2	0.056	0	0.1	0	0.000	0	0	0.009	0.186	0.775
Extremities	29	24.660	15.8	33.5	256	7.213	6.3	8.1	11	5.314	2.2	8.4	<0.001	<0.001	<0.001
Upper limb deficiency/amputation	3	2.551	0	5.4	2	0.056	0	0.1	0	0.000	0	0	<0.001	<0.001	0.090
Clubhands	2	1.701	0	4.1	2	0.056	0	0.1	0	0.000	0	0	<0.001	<0.001	0.254
Polydactyly (Hands)	3	2.551	0	5.4	13	0.366	0.2	0.6	0	0.000	0	0	0.001	0.005	0.090
Oligodactyly (Hands)	1	0.850	0	2.5	5	0.141	0	0.3	0	0.000	0	0	0.133	0.476	0.775
Adactyly (Hands)	1	0.850	0	2.5	9	0.254	0.1	0.4	0	0.000	0	0	0.344	0.748	0.775
Ectrodactyly (Hands)	1	0.850	0	2.5	1	0.028	0	0.1	0	0.000	0	0	0.001	0.080	0.775
Clinyndactyly (Hands)	0	0.000	0	0	4	0.113	0	0.2	0	0.000	0	0	0.833	1.000	NaN
Syndactyly (Hands)	0	0.000	0	0	10	0.282	0.1	0.5	0	0.000	0	0	0.633	1.000	NaN
Humerus agenesis	3	2.551	0	5.4	1	0.028	0	0.1	0	0.000	0	0	<0.001	<0.001	0.090
Radius agenesis	2	1.701	0	4.1	11	0.310	0.1	0.5	2	0.966	0	2.3	0.023	0.088	0.958
Lower limb deficiency/amputation	2	1.701	0	4.1	7	0.197	0.1	0.3	1	0.483	0	1.4	0.006	0.022	0.620
Clubfoot unilateral	5	4.252	0.5	8	63	1.775	1.3	2.2	2	0.966	0	2.3	0.094	0.110	0.122
Clubfoot bilateral	5	4.252	0.5	8	125	3.522	2.9	4.1	6	2.899	0.6	5.2	0.816	0.869	0.746
Femur agenesis	1	0.850	0	2.5	2	0.056	0	0.1	0	0.000	0	0	0.009	0.186	0.775
Sirenomelia	0	0.000	0	0	1	0.028	0	0.1	0	0.000	0	0	0.955	1.000	NaN
Tumor	0	0.000	0	0	8	0.225	0.1	0.4	0	0.000	0	0	0.694	1.000	NaN
Sacrococcygeal teratoma	0	0.000	0	0	8	0.225	0.1	0.4	0	0.000	0	0	0.694	1.000	NaN
Placenta/umbilical cord	113	96.088	79.2	112.9	2314	65.200	62.6	67.8	165	79.710	68	91.4	<0.001	<0.001	0.124
Single umbilical artery	9	7.653	2.7	12.6	281	7.918	7	8.8	19	9.179	5.1	13.3	0.815	1.000	0.799
Placentomegalia	14	11.905	5.7	18.1	44	1.240	0.9	1.6	0	0.000	0	0	<0.001	<0.001	<0.001
Placenta previa	1	0.850	0	2.5	16	0.451	0.2	0.7	2	0.966	0	2.3	0.502	1.000	1.000
Placenta previa marginal	89	75.680	60.6	90.8	1973	55.592	53.2	58	144	69.565	58.6	80.5	0.001	0.004	0.563
Amniotic fluid	48	40.816	29.5	52.1	734	20.681	19.2	22.2	47	22.705	16.3	29.1	<0.001	<0.001	0.005
Oligohydramnios	25	21.259	13	29.5	362	10.200	9.2	11.2	18	8.696	4.7	12.7	0.001	0.001	0.004
Anhydramnios	6	5.102	1	9.2	86	2.423	1.9	2.9	3	1.449	0	3.1	0.120	0.131	0.120
Polyhydramnios	17	14.456	7.6	21.3	286	8.058	7.1	9	26	12.560	7.8	17.4	0.007	0.026	0.769
Cervix <1.5cm	6	5.102	1	9.2	32	0.902	0.6	1.2	0	0.000	0	0	<0.001	<0.001	0.005

*N, number of cases; P. prevalence per 1000 pregnancies.*

#### 3.2.1 Brain abnormalities

Group I demonstrated the highest prevalence of brain abnormalities, at 74.830 per 1,000 pregnancies (95% CI: 60–90), significantly exceeding the rates in Group III (14.976 per 1,000; 95% CI: 10–20) and Group II (13.722 per 1,000; 95% CI: 13–15) (*p* < 0.001 for both comparisons). Specific conditions within this category further highlighted significant differences:

- Cerebellar hypoplasia was markedly more prevalent in Group I (4.252 per 1,000; 95% CI: 0.5–8) compared to Group II (0.141 per 1,000; 95% CI: 0–0.3) and was absent in Group III (*p* < 0.001 for Group I vs. II, *p* = 0.012 for Group I vs. III).
- Choroid plexus cysts occurred more frequently in Group I (22.959 per 1,000; 95% CI: 14.4–31.5) than in Group II (9.833 per 1,000; 95% CI: 8.8–10.9) and Group III (8.696 per 1,000; 95% CI: 4.7–12.7) (*p* < 0.001 for Group I vs. II, *p* = 0.001 for Group I vs. III).
- Holoprosencephaly showed a significantly higher prevalence in Group I (8.503 per 1,000; 95% CI: 3.3–13.8) compared to minimal occurrences in Groups II and III (*p* < 0.001 for both comparisons).
- Ventriculomegaly was observed significantly more in Group I (11.905 per 1,000; 95% CI: 5.7–18.1) than in Group II (0.225 per 1,000; 95% CI: 0.1–0.4) and Group III (0.966 per 1,000; 95% CI: 0–2.3) (*p* < 0.001 for both comparisons).

#### 3.2.2 Spine abnormalities

The prevalence of open spina bifida was highest in Group I, at 8.503 per 1,000 pregnancies (95% CI: 3.3–13.8), followed by Group II, at 2.958 per 1,000 (95% CI: 2.4–3.5), and Group III, at 1.449 per 1,000 (95% CI: 0–3.1) (*p* = 0.002 for Group I vs. II, *p* = 0.006 for Group I vs. III).

#### 3.2.3 Facial abnormalities

Cleft lip was more prevalent in Group I (7.653 per 1,000; 95% CI: 2.7–12.6) than in Groups II (0.620 per 1,000; 95% CI: 0.4–0.9) and III (0.483 per 1,000; 95% CI: 0–1.4) (*p* < 0.001 for both comparisons).

#### 3.2.4 Heart and vessels

Group I exhibited the highest prevalence of heart and vessel abnormalities at 195.578 per 1,000 pregnancies (95% CI: 172.9–218.2), significantly higher than Group III (89.855 per 1,000; 95% CI: 77.5–102.2) and Group II (71.229 per 1,000; 95% CI: 68.6–73.9) (*p* < 0.001 for both comparisons). Specific conditions within this category further highlighted significant differences:

- Tricuspid Regurgitation: This condition was more prevalent in Group I, observed in 11.905 per 1,000 pregnancies (95% CI: 5.7–18.1), in contrast to 4.170 in Group II (95% CI: 3.5–4.8) and 3.382 in Group III (95% CI: 0.9–5.9) (*p* < 0.001 for Group I vs. II, *p* = 0.007 for Group I vs. III).
- Supraventricular Arrhythmia: The prevalence was notably higher in Group I at 21.259 per 1,000 pregnancies (95% CI: 13–29.5), compared to 1.324 in Group II (95% CI: 0.9–1.7) and 1.449 in Group III (95% CI: 0–3.1) (*p* < 0.001 for both comparisons).
- Ventricular Septal Defect (VSD): While Group I had a prevalence of 19.558 per 1,000 (95% CI: 11.6–27.5), Group II recorded 8.340 per 1,000 (95% CI: 7.4–9.3), and Group III had the highest among the non-Group I populations at 27.053 per 1,000 (95% CI: 20.1–34) (*p* < 0.001 for Group I vs. II, *p* = 0.225 for Group I vs. III).
- Transposition of the Great Arteries (TGA): Group I had a significantly higher prevalence at 5.952 per 1,000 pregnancies (95% CI: 1.6–10.3) compared to Group II at 1.324 per 1,000 (95% CI: 0.9–1.7) and Group III at 0.966 per 1,000 (95% CI: 0–2.3) (*p* < 0.001 for Group I vs. II, *p* = 0.025 for Group I vs. III).
- Tetralogy of Fallot (ToF): Found in 2.551 per 1,000 pregnancies (95% CI: 0–5.4) in Group I, 1.240 per 1,000 (95% CI: 0.9–1.6) in Group II, and 0.483 per 1,000 (95% CI: 0–1.4) in Group III (*p* = 0.411 for Group I vs. II, *p* = 0.274 for Group I vs. III).
- Aortic Coarctation: The prevalence of aortic coarctation in Group I was 2.551 per 1,000 (95% CI: 0–5.4), compared to 0.423 (95% CI: 0.2–0.6) in Group II and 0.483 (95% CI: 0–1.4) in Group III (*p* = 0.010 for Group I vs. II, *p* = 0.274 for Group I vs. III).
- Hypoplastic Left Heart Syndrome (HLHS): More prevalent in Group I (4.252 per 1,000; 95% CI: 0.5–8) than in Group II (1.437 per 1,000; 95% CI: 1–1.8) and absent in Group III (*p* = 0.040 for Group I vs. II, *p* = 0.012 for Group I vs. III).

#### 3.2.5 Abdominal wall

Gastroschisis had a high prevalence in Group I at 26.361 per 1,000 (95% CI: 17.2–35.5), compared to Group II at 1.155 per 1,000 (95% CI: 0.8–1.5) and no cases in Group III (*p* < 0.001 for both comparisons).

#### 3.2.6 Urinary tract

The prevalence of urinary tract abnormalities was significantly higher in Group I at 52.721 per 1,000 (95% CI: 39.9–65.5) compared to Group II at 16.229 per 1,000 (95% CI: 14.9–17.5) and Group III at 13.043 per 1,000 (95% CI: 8.2–17.9) (*p* < 0.001 for both comparisons).

#### 3.2.7 Extremities

Limb deficiencies were more prevalent in Group I at 24.660 per 1,000 (95% CI: 15.8–33.5) compared to Group II at 7.213 per 1,000 (95% CI: 6.3–8.1) and Group III at 5.314 per 1,000 (95% CI: 2.2–8.4) (*p* < 0.001 for both comparisons).

#### 3.2.8 Thorax abnormalities

Diaphragmatic hernia was more prevalent in Group I at 5.102 per 1,000 (95% CI: 1–9.2) than in Group II at 2.029 (95% CI: 1.6–2.5) and Group III at 1.449 (95% CI: 0–3.1) (*p* = 0.054 for Group I vs. II, *p* = 0.120 for Group I vs. III).

#### 3.2.9 Genital tract abnormalities

Genital tract abnormalities were more common in Group I at 5.102 (95% CI: 1–9.2) compared to Group II at 0.423 (95% CI: 0.2–0.6), with no cases in Group III (*p* < 0.001 for both comparisons).

#### 3.2.10 Other observations

Amniotic fluid abnormalities, such as oligohydramnios (21.259 per 1,000; 95% CI: 13–29.5) and polyhydramnios (14.456 per 1,000; 95% CI: 7.6–21.3), were significantly more prevalent in Group I compared to Groups II and III (*p* = 0.001 for Group I vs. II, *p* = 0.004 for Group I vs. III).

### 3.3 Risk ratios for fetal abnormalities in adolescent pregnancy

The relative risk of any fetal abnormalities detected during a mid-trimester ultrasound in pregnant adolescents (14–19 years old) compared to adult pregnant women in reference group (20–39 years old) is 12.51(95% CI: 10.67–14.65).

#### 3.3.1 Abdominal wall and gastrointestinal tract abnormalities

The risk of abdominal wall abnormalities is associated with a risk ratio of 9.72 (95% CI: 6.79–13.91). Among these, the risk of gastroschisis is the highest, with an RR of 22.90 (95% CI: 14.46–36.25). Omphalocele is associated with an RR of 3.12 (95% CI: 1.47–6.60). For body-stalk anomaly, an RR of 2.32 (95% CI: 0.13–41.17) was observed.

Regarding gastrointestinal tract abnormalities, the analysis showed that the overall risk for any abnormality in this category remains inconclusive, with an RR of 0.89 (95% CI: 0.47–1.69). However, a significantly increased risk was found for esophageal atresia, with an RR of 34.81 (95% CI: 12.20–99.34). Small bowel obstruction was associated with an RR of 7.94 (95% CI: 1.97–31.94). In contrast, a lower risk was observed for hyperechogenic bowel, with an RR of 0.06 (95% CI: 0.00–0.96). The analyses for duodenal atresia and ascites yielded inconclusive results, with RRs of 0.77 (95% CI: 0.05–12.80) and 0.40 (95% CI: 0.02–6.55), respectively.

**Figure 1:**
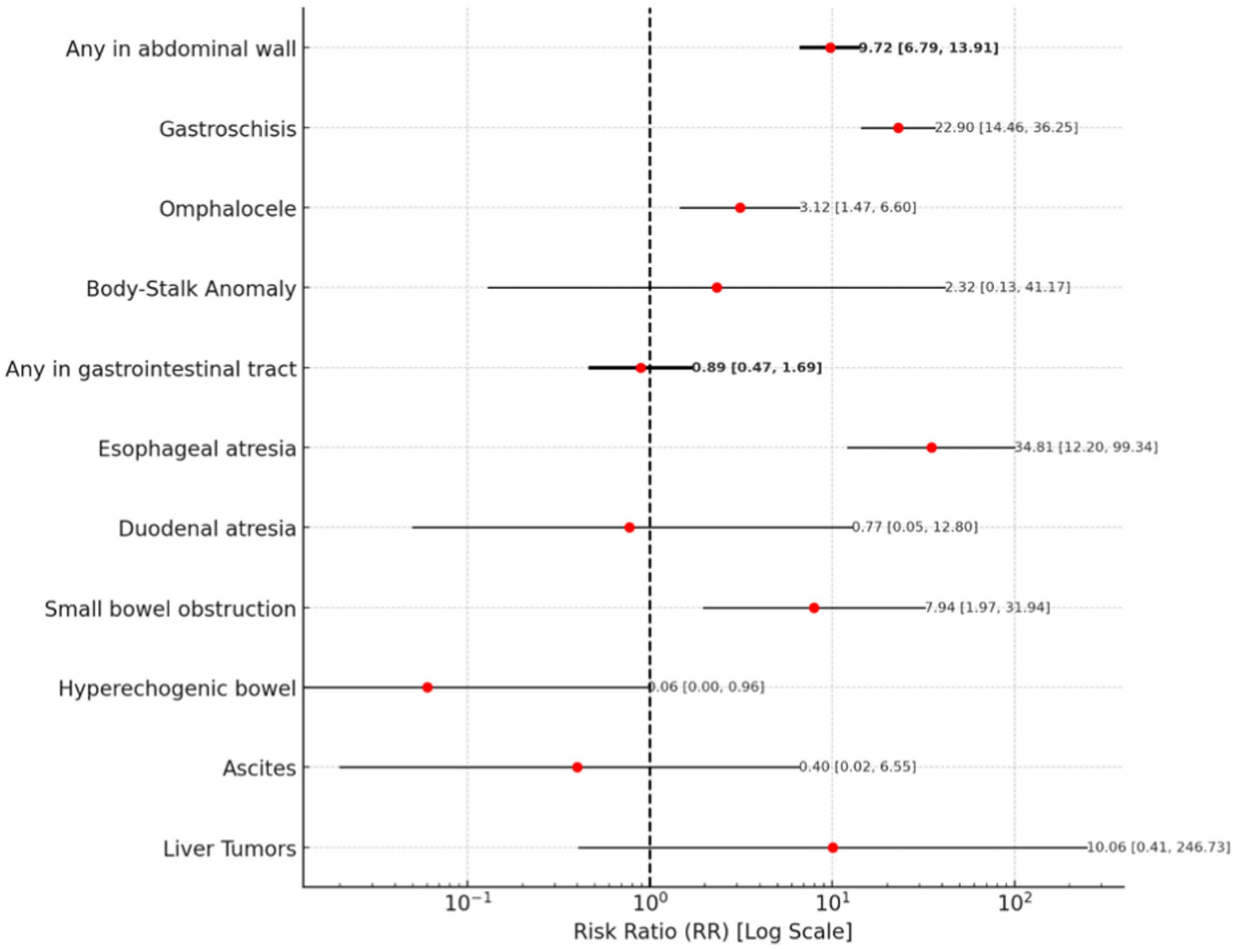
A forest plot for abdominal wall and gastrointestinal tract abnormalities.

A forest plot illustrating abdominal wall and gastrointestinal tract abnormalities is presented in Figure 1.

#### 3.3.2 Brain and spinal abnormalities

The risk of brain abnormalities is associated with a risk ratio of 5.48 (95% CI: 4.40–6.82). Holoprosencephaly has an exceptionally high RR of 211.17 (95% CI: 38.20–1167.36). Ventriculomegaly exhibits a RR of 51.46 (95% CI: 22.11–119.76), while cerebellar hypoplasia and Blake’s pouch cyst are both associated each with an RR of 30.17 (95% CI: 9.27–98.22 and 5.23–173.97). Corpus callosum agenesis and mega cisterna magna show comparable RRs of 26.62 (95% CI: 9.99–70.94) and 24.68 (95% CI: 7.11–85.68). Microcephaly and craniosynostosis are also associated with an increased risk, with RRs of 17.05 (95% CI: 6.53–44.53) and 12.93 (95% CI: 1.91–87.47), respectively. Dolichocephaly has an RR of 8.97 (95% CI: 3.47–23.19), while acrania is associated with an RR of 7.34 (95% CI: 2.62–20.52). Dandy-Walker Syndrome carries an RR of 5.59 (95% CI: 1.45–21.51), and open spina bifida presents an elevated risk with an RR of 3.00 (95% CI: 1.60–5.65).

Some results remain inconclusive; for instance, the RRs for brachycephaly and sacrococcygeal teratoma are 0.97 (95% CI: 0.19–4.94) and 1.77 (95% CI: 0.10–30.73), respectively.

**Figure 2:**
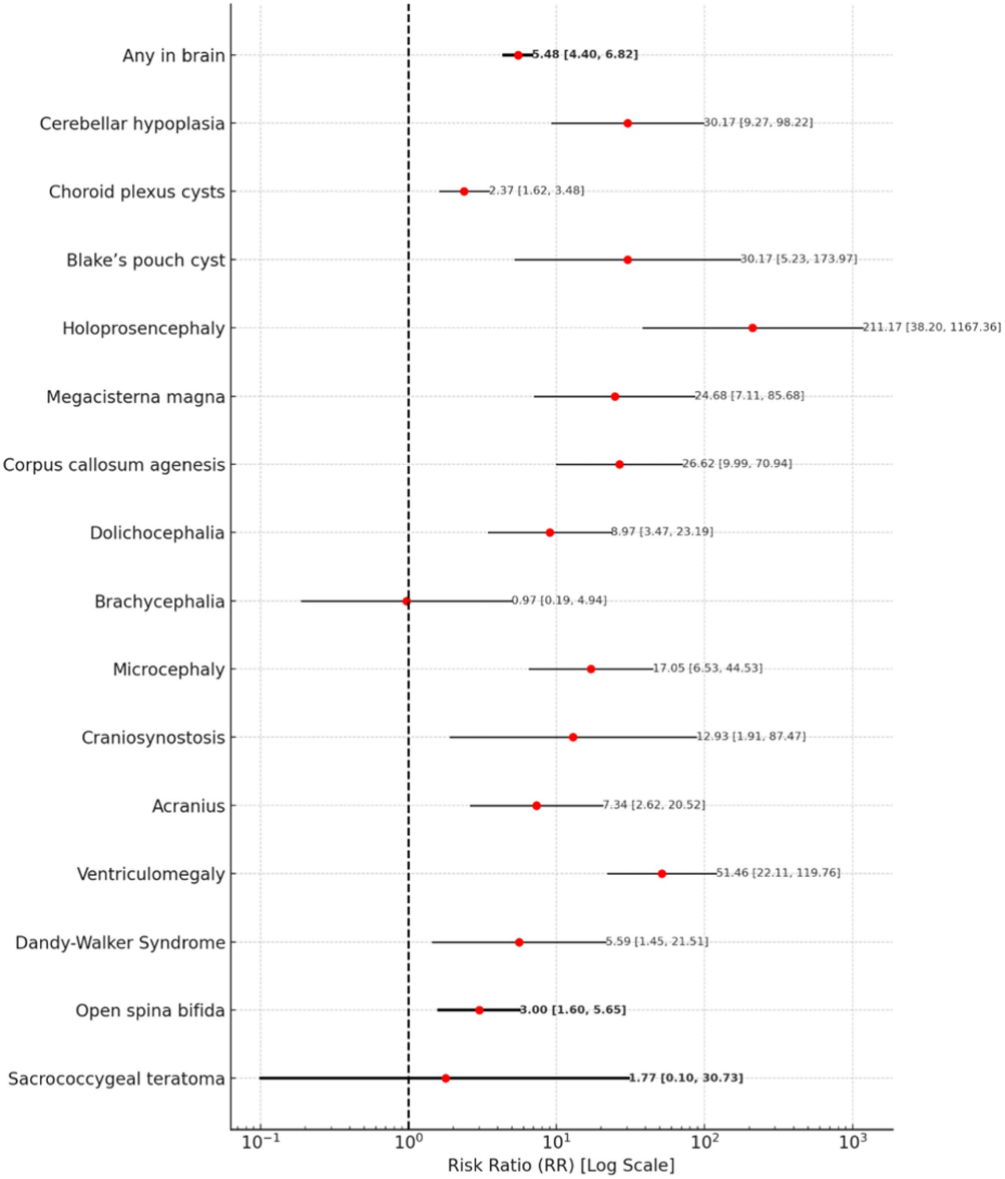
A forest plot for brain and spinal abnormalities.

A forest plot illustrating brain and spinal abnormalities is presented in Figure 2.

#### 3.3.3 Extremities abnormalities

The risk of extremity abnormalities is associated with a risk ratio of 3.47 (95% CI: 2.38–5.06).

For upper limb abnormalities, humerus agenesis carries the highest risk, with an RR of 70.39 (95% CI: 10.40–476.24), followed by upper limb deficiency or amputation (RR = 42.23, 95% CI: 8.34–213.84). Clubhands and ectrodactyly both have an RR of 30.17, with confidence intervals of 5.23–173.97 and 3.14–289.81, respectively. Polydactyly has an RR of 7.82 (95% CI: 2.42–25.31), while oligodactyly presents an RR of 8.23 (95% CI: 1.35–50.00). Radius agenesis has an RR of 6.56 (95% CI: 1.67–25.72).

For lower limb abnormalities, femur agenesis is associated with a highest RR of 18.10 (95% CI: 2.39–136.92), while lower limb deficiency or amputation has an RR of 10.06 (95% CI: 2.41–42.03). Sirenomelia presents an RR of 10.06 (95% CI: 0.41–246.73). In contrast, bilateral clubfoot has a much lower risk, with an RR of 1.32 (95% CI: 0.56–3.10).

**Figure 3:**
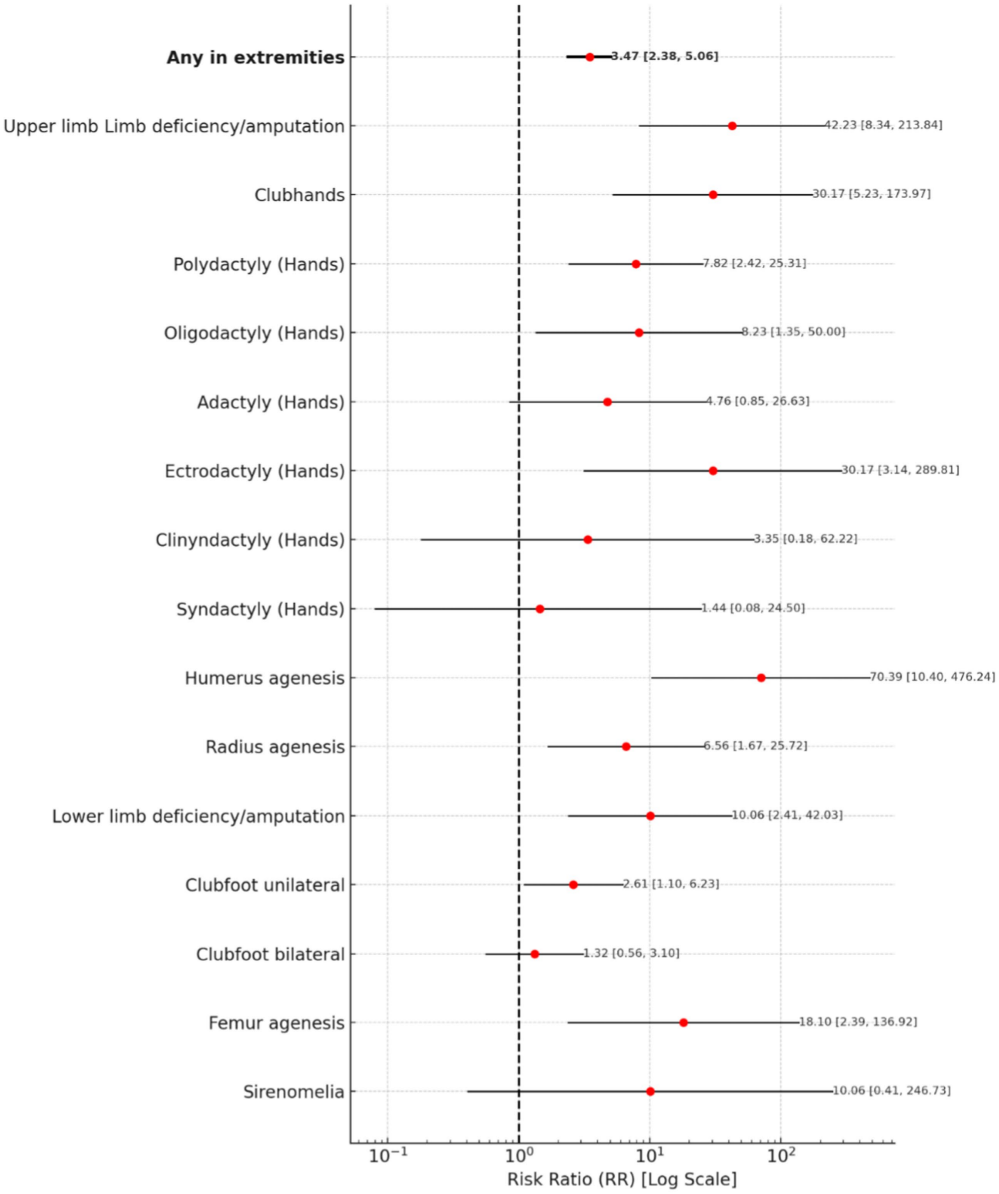
A forest plot for extremities abnormalities.

A forest plot for Extremities Abnormalities is presented in Figure 3.

#### 3.3.4 Face, neck, and thorax abnormalities

The risk of any facial abnormality is associated with a risk ratio of 2.74 (95% CI: 1.86–4.03). Cleft lip carries a notably high risk, with an RR of 12.74 (95% CI: 5.98–27.13). Cleft lip and palate are also associated with an RR of 2.29 (95% CI: 1.04–5.08), while hypertelorism indicates an RR of 3.87 (95% CI: 1.04–14.41). Micrognathia has an RR of 2.29 (95% CI: 0.96–5.43), while nasal bone hypoplasia presents an RR of 1.33 (95% CI: 0.46–3.87).

The overall risk of any neck abnormality remains with an RR of 1.85 (95% CI: 0.71–4.78). Cystic hygroma has an RR of 1.35 (95% CI: 0.26–6.93), whereas nuchal edema presents an RR of 2.82 (95% CI: 0.94–8.41). Cervical teratoma shows an inconclusive risk, with an RR of 4.31 (95% CI: 0.22–83.39), while cervical lymphangioma presents an RR of 2.74 (95% CI: 0.15–49.57).

The risk of any thoracic abnormality is associated with a risk ratio of 2.25 (95% CI: 1.24–4.08). Diaphragmatic hernia presents an increased risk, with an RR of 2.70 (95% CI: 1.21–6.02). Congenital pulmonary airway malformation indicates an RR of 3.70 (95% CI: 1.22–11.23). Pleural effusion has an RR of 1.21 (95% CI: 0.24–6.16), while pulmonary sequestration presents an RR of 1.77 (95% CI: 0.10–30.73).

**Figure 4:**
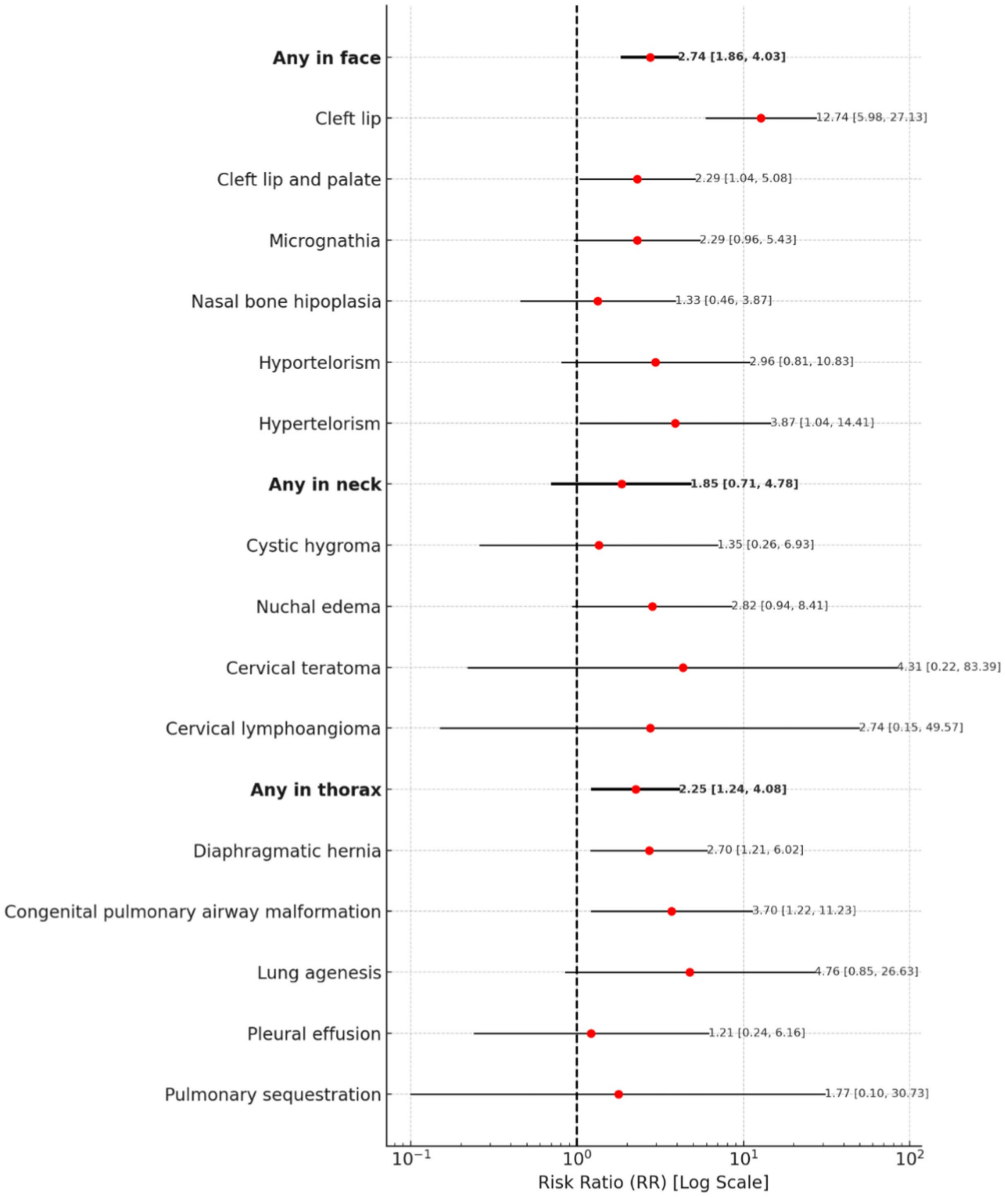
A forest plot for face, neck, and thorax abnormalities.

A forest plot for Face, Neck, and Thorax Abnormalities is presented in Figure 4.

#### 3.3.5 Heart and vessels abnormalities

The risk of any heart or vessel abnormality is associated with a risk ratio (RR) of 2.75 (95% CI: 2.43–3.11). Among the specific abnormalities, persistent right umbilical vein (PRUV) presents the highest risk, with an RR of 271.50 (95% CI: 14.63–5040.15), followed by persistent left superior vena cava (PLSVC) with an RR of 150.84 (95% CI: 7.25–3140.23). Agenesis of the ductus venosus also shows a notably high risk, with an RR of 47.41 (95% CI: 12.43–180.85). Supraventricular arrhythmia has an RR of 16.19 (95% CI: 10.04–26.11), while aortic valve atresia and interrupted aortic arch (IAA) present significantly increased risks, with RRs of 13.52 (95% CI: 5.37–34.05) and 13.71 (95% CI: 3.08–61.08), respectively. Tachycardia shows an RR of 11.95 (95% CI: 5.86–24.37), while both heart ectopy and hypoplastic right heart syndrome (HRHS) present an RR of 10.06 (95% CI: 0.41–246.73) and (95% CI: 1.59–63.76).

Several other abnormalities also indicate a notably increased risk. Hypertrophic cardiomyopathy and agenesis of the inferior vena cava both have an RR of 7.94 (95% CI: 1.97–31.94). Aberrant right subclavian artery (ARSA) presents an RR of 7.76 (95% CI: 2.76–21.82), and right aortic arch (RAA) is associated with an RR of 7.18 (95% CI: 1.81–28.50).

Some results remain inconclusive. Double inlet left ventricle (DILV) shows an RR of 3.35 (95% CI: 0.18–62.22), while double outlet right ventricle (DORV) has an RR of 3.21 (95% CI: 0.87–11.81). Pulmonary atresia (PA) and tricuspid valve atresia both present an RR of 3.12 (95% CI: 0.58–16.75). Tetralogy of Fallot (ToF) has an RR of 2.37 (95% CI: 0.80–7.03).

Other congenital defects with inconclusive risk estimates include truncus arteriosus communis (TAC) with an RR of 1.31 (95% CI: 0.08–22.25), and Ebstein’s anomaly, hypoplastic aortic arch, and single ventricle defect, each with an RR of 1.59 (95% CI: 0.09–27.26). Atrioventricular septal defect (AVSD) presents an RR of 1.10 (95% CI: 0.31–3.88), while mitral regurgitation has an RR of 1.31 (95% CI: 0.08–22.25). Finally, situs inversus presents an RR of 1.12 (95% CI: 0.07–18.78).

**Figure 5:**
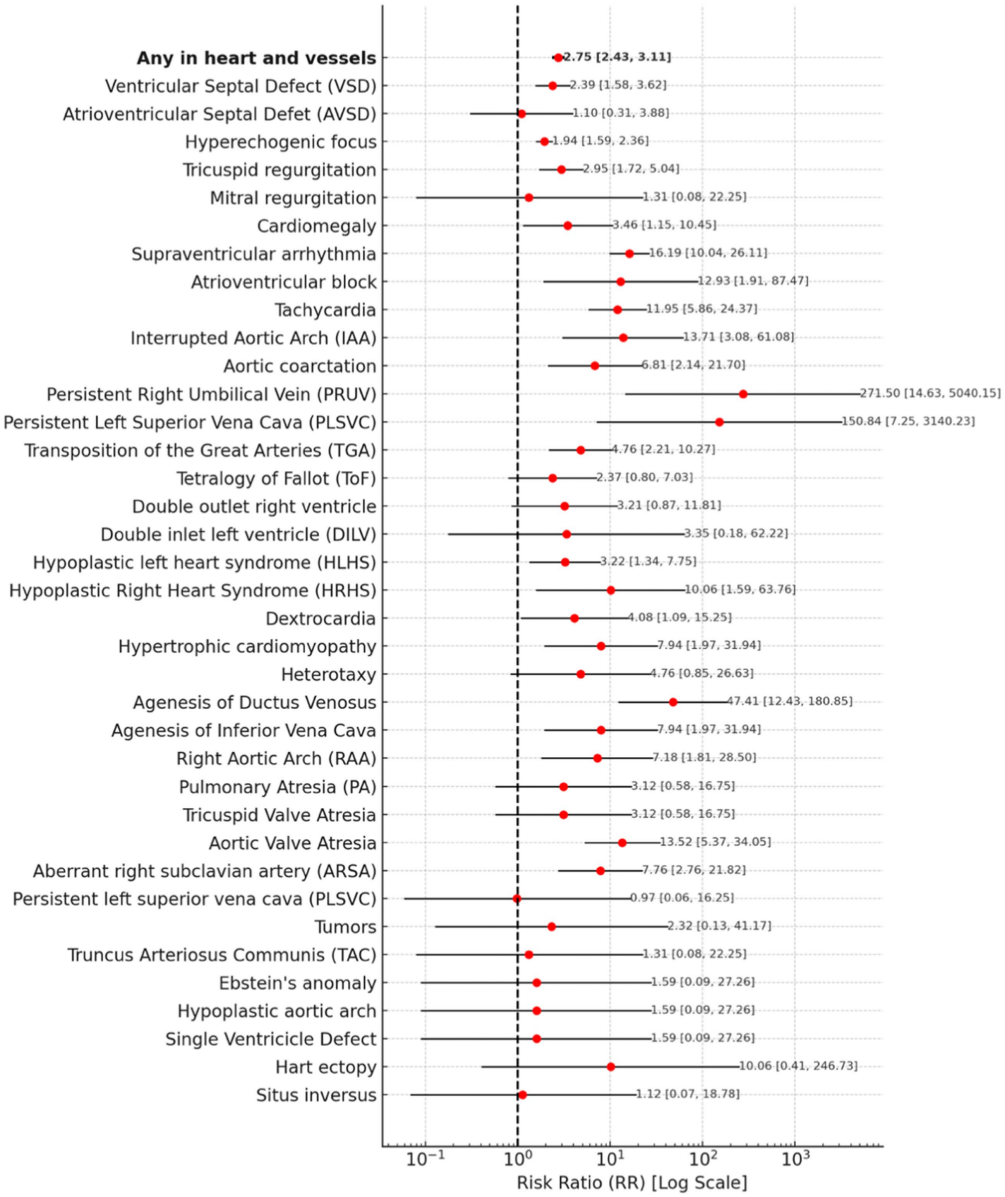
A forest plot for heart and vessels abnormalities.

A forest plot for Heart and vessels abnormalities is presented in Figure 5.

#### 3.3.6 Urinary and genital tract abnormalities

The risk of any urinary tract abnormality is associated with a risk ratio of 3.27 (95% CI: 2.54–4.22).

Among specific conditions, autosomal dominant polycystic kidney disease presents the highest risk, with an RR of 392.17 (95% CI: 22.10–6957.71). Autosomal recessive polycystic kidney disease also shows a markedly high RR of 90.50 (95% CI: 3.69–2220.53). Bladder exstrophy presents an RR of 50.28 (95% CI: 6.65–380.32). Megacystis is associated with an RR of 12.42 (95% CI: 3.58–43.07), while pyelectasis indicates an increased risk with an RR of 5.51 (95% CI: 2.66–11.43).

Some results remain inconclusive. Polycystic kidneys show an RR of 0.34 (95% CI: 0.02–5.50), and multicystic kidneys have an RR of 0.23 (95% CI: 0.01–3.72).

The risk of any genital tract abnormality is significantly increased, with an RR of 12.65 (95% CI: 5.07–31.56). Among specific conditions, hypospadias presents the highest risk, with an RR of 18.10 (95% CI: 2.39–136.92) and ovarian cysts indicate risk, with an RR of 12.29 (95% CI: 4.57–33.07).

**Figure 6:**
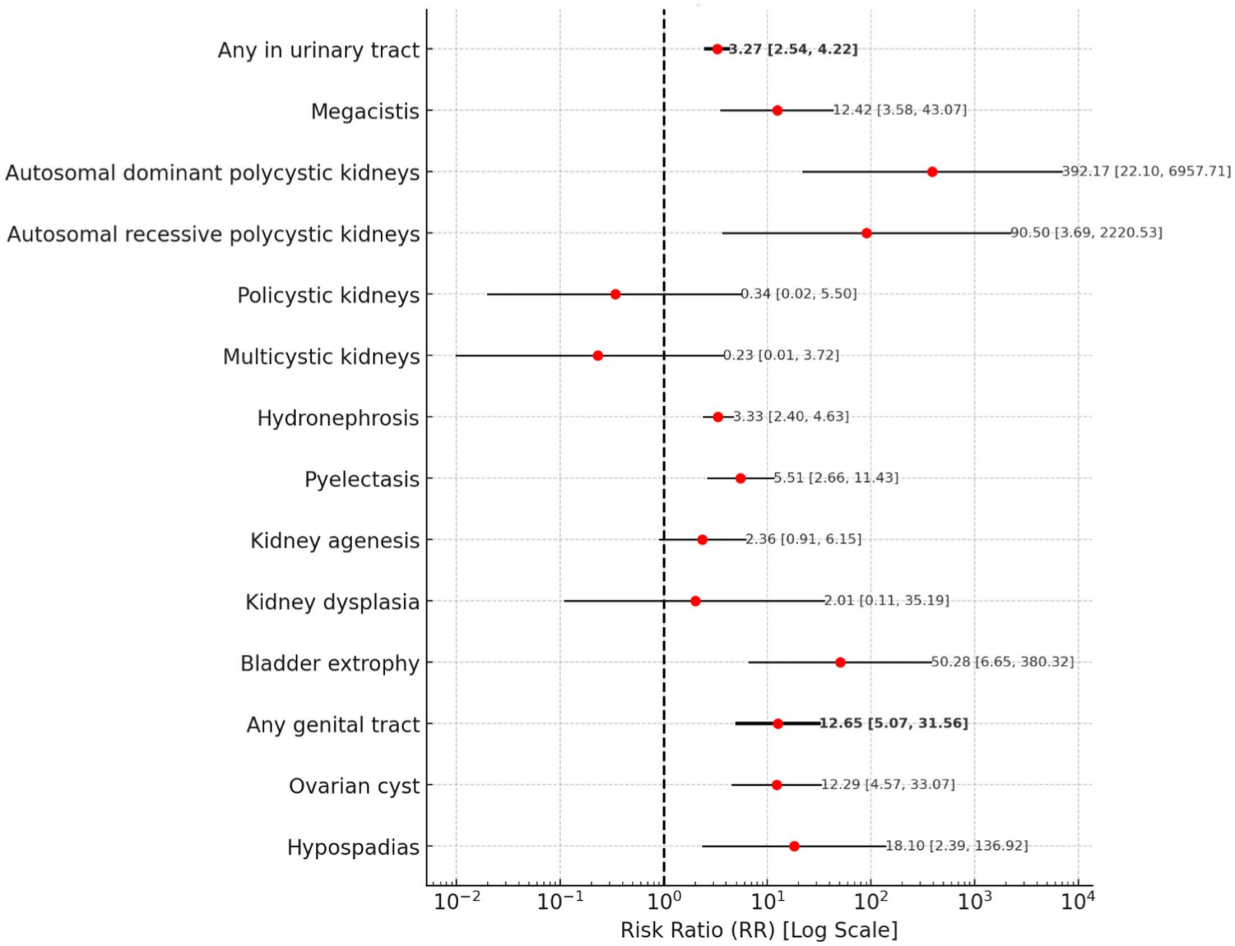
A forest plot for urinary and genital tract abnormalities.

Figure 6 presents a forest plot for Urinary and Genital Tract Abnormalities.

#### 3.3.7 Placenta, amniotic fluid and cervical abnormalities

The risk of any placental or umbilical cord abnormality is associated with a risk ratio of 1.48 (95% CI: 1.24–1.77). Among specific conditions, placentomegaly presents the highest risk, with an RR of 9.83 (95% CI: 5.45–17.73). Placenta previa has risk, with an RR of 2.74 (95% CI: 0.52–14.58), while marginal placenta previa shows an RR of 1.37 (95% CI: 1.12–1.68).

For amniotic fluid abnormalities, the presence of any abnormality is associated with an RR of 1.99 (95% CI: 1.50–2.65). Anhydramnios presents an increased risk, with an RR of 2.27 (95% CI: 1.02–5.02), followed by oligohydramnios, which has an RR of 2.12 (95% CI: 1.43–3.16). Polyhydramnios indicates RR of 1.84 (95% CI: 1.14–2.98).

Regarding cervical abnormalities, a cervical length of less than 15 mm is associated with a increased risk, with an RR of 6.03 (95% CI: 2.60–13.98).

**Figure 7:**
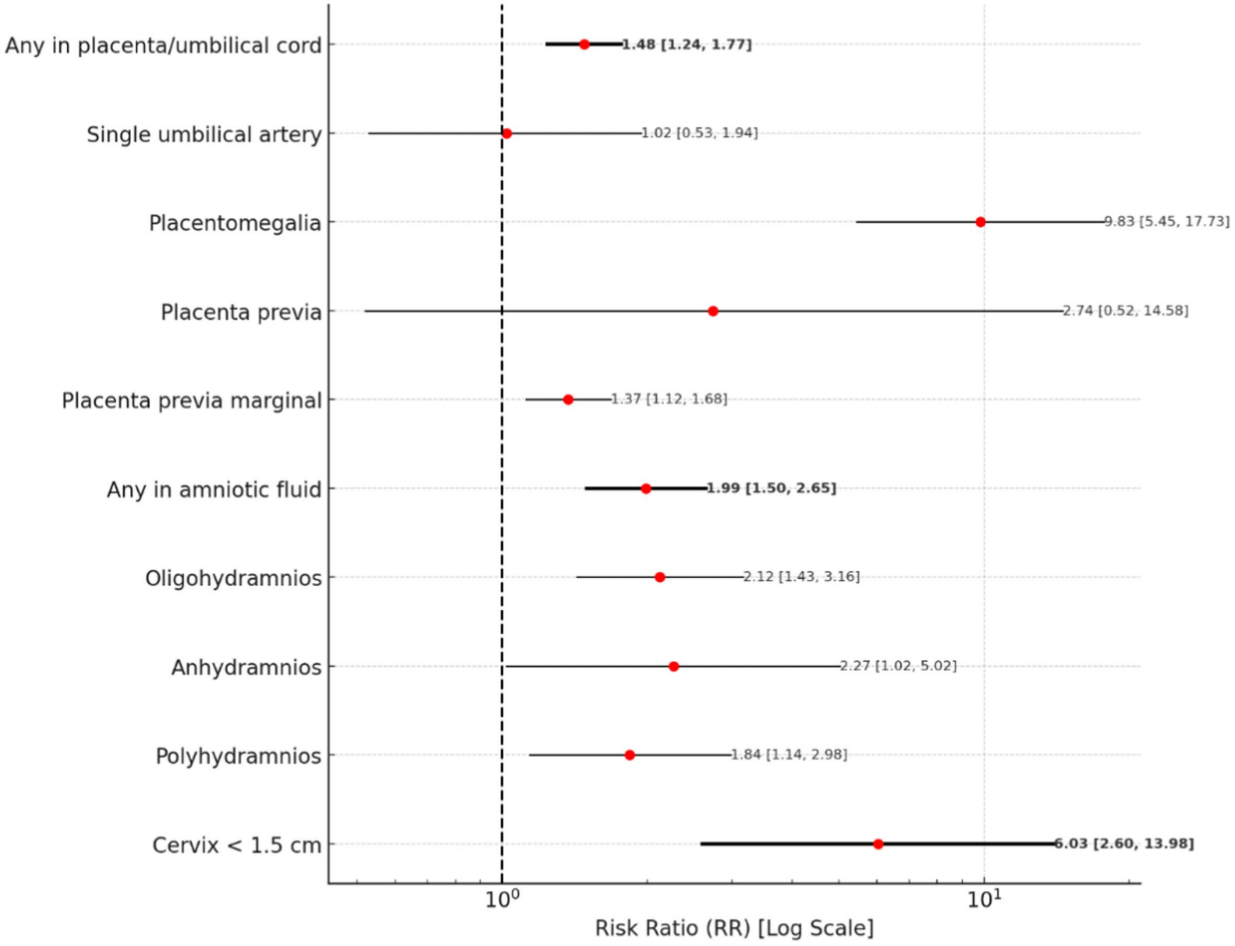
A forest plot for placenta and amniotic fluid abnormalities.

A forest plot for Placenta, Amniotic Fluid and Cervical Abnormalities is presented in Figure 7.

## 4 Discussion

This study highlights significant differences in prevalence of ultrasonographic findings in mid-trimester scans between adolescent pregnancies and those in older women. Our results indicate that adolescent pregnancies have a markedly higher prevalence of fetal abnormalities in mid-trimester US scan, with a prevalence rate of 437.075 per 1,000 pregnancies (95% CI: 409–465), significantly higher than in older age groups. The overall risk of detecting fetal abnormalities during mid-trimester ultrasounds is substantially higher in adolescent pregnancies, with a relative risk (RR) of 12.51 (95% CI: 10.67–14.65).

Our findings align with previous studies reporting increased risks of congenital anomalies in younger mothers. For instance, Reefhuis et al. (18) found that both teenage and older maternal age groups are at higher risk for non-chromosomal birth defects compared to women in their twenties. Similarly, Gill et al. (19) noted an association between younger maternal age and birth defects of unknown etiology, highlighting the vulnerability of this demographic. Additionally, Loane et al. (3) observed that younger maternal age is associated explicitly with a higher risk of non-chromosomal anomalies, corroborating our findings of increased abnormalities, especially in abdominal wall defects.

Our study reveals a significantly higher prevalence of gastroschisis in adolescent pregnancies compared to older age groups. Previous research, such as the studies by Benjamin et al. (20), Torfs et al. (21), and Chabra et al. (22), supports our findings. Benjamin et al. documented the prevalence of gastroschisis in Texas from 1999 to 2003, highlighting a higher incidence in younger mothers compared to older ones. Chabra et al. reported a rising prevalence of gastroschisis in Washington State, indicating that this condition is becoming more common, particularly among young mothers. Torfs et al., a case–control study conducted within the population surveyed by the California Birth Defects Monitoring Program (CBDMP), also found a significant correlation between young maternal age and gastroschisis, a congenital abdominal wall defect. Csermely et al. (23), Reefhuis et al. (18), and Gill et al. (19) support these conclusions, emphasizing the overall increased risk of congenital defects in younger mothers.

A recent systematic review by Pethő et al. (24), based on 72 studies, stated that young maternal age (14–19 years) is associated with increased risks of anencephaly and hydrocephaly. Our study found the prevalence of brain abnormalities in adolescent pregnancies to be 74.830 per 1,000 pregnancies (95% CI: 60–90), notably higher than in older groups. Xi-Kuan Chen et al. (4) also found that teenage pregnancies are associated with a higher risk of central nervous system anomalies. Our results are consistent with these data, notably showing exceptionally high risks for holoprosencephaly (RR: 211.17, 95% CI: 38.20–1167.36) and ventriculomegaly (RR: 51.46, 95% CI: 22.11–119.76).

Our study demonstrates a significantly elevated risk of congenital limb defects in adolescent pregnancies, with a relative risk (RR) of 3.47 (95% CI: 2.38–5.06). Notably, conditions such as upper limb deficiency/amputation (RR: 42.23, 95% CI: 8.34–213.84), clubhands (RR: 30.17, 95% CI: 5.23–173.97), and humeral agenesis (RR: 70.39, 95% CI: 10.40–476.24) were particularly prevalent. These findings are corroborated by Pethő et al. (18), who confirm that young maternal age significantly increases the risk of congenital limb defects. Similarly, Reefhuis (18) identified a higher risk of polydactyly (OR = 1.29) in younger mothers. In addition to limb defects, our research highlights a substantially increased risk of facial defects in adolescent pregnancies, with an RR of 2.74 (95% CI: 1.86–4.03). Specific conditions such as cleft lip (RR: 12.74, 95% CI: 5.98–27.13) and cleft lip and palate (RR: 2.29, 95% CI: 1.04–5.08) were particularly significant. These results align with Reefhuis (18), who reported an increased risk of cleft lip (OR = 1.88) in younger age groups, and Pethő et al. (24), who confirmed that both very young and advanced maternal ages are associated with a higher risk of facial defects.

Our study reveals a significantly increased risk of heart and vascular defects in adolescent pregnancies (RR: 2.75, 95% CI: 2.43–3.11), with specific conditions such as ductus venosus agenesis (RR: 47.41, 95% CI: 12.43–180.85) and supraventricular arrhythmia (RR: 16.19, 95% CI: 10.04–26.11) being prominently noted. These findings are supported by Pethő et al. (24), who indicate a high risk of heart defects in very young mothers, and by Csermely et al. (23), who noted an increased risk of heart defects, particularly left-sided obstructive defects. Furthermore, the study indicates a significantly increased risk of urinary system defects in adolescent pregnancies (RR: 3.27, 95% CI: 2.54–4.22), with extreme risks observed for autosomal dominant polycystic kidney disease (RR: 392.17, 95% CI: 22.10–6957.71) and bladder exstrophy (RR: 50.28, 95% CI: 6.65–380.32). These findings are consistent with Reefhuis (18), who reported a higher risk of hydronephrosis (OR = 1.42) and female genital defects (OR = 1.57) in fetuses of younger mothers, as well as Csermely et al. (23), who noted a higher risk of undescended testes in younger mothers.

A significant number of congenital defects in adolescent patients have been estimated at 437.075 per 1,000 pregnancies (95% CI: 409–465). Some studies suggest that this may be attributed to delayed disclosure of pregnancy, as demonstrated by the low percentage of 1st trimester US carried out in adolescents in comparison to older pregnancies. Pregnancy at a young age is often associated with shame and the stigma of being pregnant, which often leads to delays in informing parents about the possibility of pregnancy (1). This postpones confirmation of the pregnancy by a doctor and can negatively impact both the pregnancy and the developing fetus. Patients under 19 years old presented for second-trimester screening 2 weeks and 2 days later than older women. Notably, only 29.08% of patients under 19 years old had prenatal screening in the first trimester, which could have detected abnormalities earlier. The younger the patients, the less likely they were to have first-trimester screening: 20.9% of 17-year-old patients, 18.2% of 16-year-olds, 11.8% of 15-year-olds, and even less frequently among 14-year-olds. The lack of first-trimester prenatal screening and late second-trimester screening practically excluded the possibility of pregnancy termination due to fetal defects. Additionally, the high level of abnormalities in adolescent patients can also be explained by the fact that underage patients were referred for screening only when a gynecologist noticed abnormalities; otherwise, they would not have been referred. During the study period, ultrasound examination reimbursement under Polish law was granted to patients over 35 years of age, if chromosomal aberrations occurred in a previous pregnancy, if chromosomal aberrations were found in the mother or father of the unborn child, in situations where there was an increased risk that the child would be born with a monogenetically or multifactorial conditioned disease, or if ultrasound or biochemical analyses indicated the possibility of defects or chromosomal aberrations in the fetus.

Later recognition of the pregnancy can also result in the lack of supplementation and the continuation of substance use. Torf et al. (21) asserted that young maternal age is consistently identified as a significant risk factor for gastroschisis, primarily due to substance use during pregnancy, including smoking, alcohol consumption, and illicit drug use. It is important to note that the occurrence of fetal anomalies may be associated with the low socioeconomic status of adolescent pregnancies. Socioeconomic factors such as low income and lower educational levels are also associated with an increased risk of abdominal wall defects.

Adolescent pregnancy is linked with significant nutritional risks, with studies demonstrating inadequate intake of essential nutrients such as folate, iron, and vitamin B12 (25, 26). Folate deficiency is particularly concerning, with high prevalence rates observed in pregnant adolescents. Reis et al. found that adolescent pregnancy has a higher prevalence of neural tube defects, likely due to a lack of folic acid supplementation (6). Multiple micronutrient deficiencies often coexist, and low folate levels are significantly associated with anemia (27). Socioeconomic factors, dietary intake, and smoking habits influence micronutrient status (25). Adolescent pregnancy is also associated with high rates of substance use, which poses significant health risks. Studies report prevalence rates of 17–75.5% for alcohol use, 17–27.8% for smoking, and 1.7–28.57% for illicit drug use among pregnant adolescents (28, 29). Reports indicate that 33.4% of adolescents engage in heavy episodic alcohol consumption and that 34.8% of adolescents are sexually active by the age of 15 without using any form of contraception. Combined, these behaviors can lead to adolescent pregnancies complicated by fetal alcohol syndrome (FAS) (30).

In conclusion, young maternal age is associated with increased detection of various fetal abnormalities during mid-trimester ultrasound scans. Contrary to common belief, the young age of adolescent pregnant individuals does not protect against fetal abnormalities. These findings underscore the necessity for comprehensive, population-based ultrasound screening for pregnant adolescents and the classification and management of adolescent pregnancies as high-risk.

## 5 Limitations

The limitations of this study include the fact that data were collected from outpatient referral centers, where many patients sought care exclusively for ultrasound examinations and gave birth in other facilities. As a result, postnatal outcome data were not included, as the authors often lacked access to such information. Legal constraints further restricted access to postnatal data, making it unfeasible to collect comprehensive follow-up information. Therefore, the primary aim of this study was limited to assessing ultrasound findings during mid-trimester examinations, which restricts the ability to establish correlations between prenatal diagnoses and postnatal outcomes.

Moreover, some data, such as those related to assisted reproductive technology (ART) and the occurrence of multiple pregnancies, were too limited to allow for additional statistical analyses, representing another study limitation. However, existing literature suggests that these factors may contribute to a higher incidence of congenital heart defects in monochorionic twins conceived via ART compared to those conceived naturally (31).

Another limitation is that adolescent pregnant women were referred for mid-trimester ultrasound only when abnormalities were suspected by a gynecologist. Otherwise, they were not routinely screened. Furthermore, during the study period, Polish law provided ultrasound reimbursement only under specific conditions, primarily for patients over 35 years of age or those with an elevated risk of genetic disorders, which may have influenced the composition of the study cohort.

Additionally, the study group was ethnically homogeneous, predominantly white, which may limit the generalizability of the findings to more diverse populations. Finally, advances in ultrasound technology have improved the detection of abnormalities, potentially contributing to variability in results depending on the period during which the examinations were performed.

## 6 Clinical implications

Given the higher prevalence of fetal abnormalities in mid-trimester ultrasound scans in adolescent pregnancies, there is a clear need for enhanced prenatal screening protocols tailored specifically for this age group. Early and comprehensive prenatal screening can help timely detect and manage fetal anomalies, thereby improving pregnancy outcomes. Moreover, the role of public education and the involvement of parents of teenage pregnant women is crucial in ensuring that patients are not reluctant to seek medical advice at an early stage of pregnancy. By emphasizing the importance of early medical consultations through targeted educational campaigns and parental support, healthcare providers can foster an environment where adolescent patients feel empowered to access necessary prenatal care, ultimately leading to better health outcomes for both the mother and the fetus (32).

## Data Availability

The raw data supporting the conclusions of this article will be made available by the authors, without undue reservation.
