# Supplementary material for: Ultrasonographic findings in mid-trimester adolescent pregnancy: prevalence and risk of abnormalities
Source: Front Med (Lausanne). 2025 Apr 30;12:1525149. doi: 10.3389/fmed.2025.1525149 (PMC12074930; doi:10.3389/fmed.2025.1525149)
Supplement: Supplementary file 1 [file Supplementary_file_1.docx]

Appendix 1

A detailed characteristics of the participants.

| **Patient Age (Years)** | **14** | **15** | **16** | **17** | **18** | **19** | **20** | **21** | **22** | **23** | **24** | **25** | **26** | **27** | **28** | **29** | **30** | **31** | **32** | **33** | **34** | **35** | **36** | **37** | **38** | **39** | **40** | **41** | **42** | **43** | **44** | **45** | **46** | **47** |
| --- | --- | --- | --- | --- | --- | --- | --- | --- | --- | --- | --- | --- | --- | --- | --- | --- | --- | --- | --- | --- | --- | --- | --- | --- | --- | --- | --- | --- | --- | --- | --- | --- | --- | --- |
| **Patients (N)** | 41 | 68 | 154 | 268 | 301 | 320 | 404 | 527 | 700 | 930 | 1224 | 1634 | 1988 | 2361 | 2448 | 2430 | 2449 | 2237 | 1952 | 1859 | 1871 | 2661 | 2241 | 1815 | 1367 | 1094 | 798 | 599 | 304 | 174 | 83 | 39 | 19 | 6 |
| **GA** |  |  |  |  |  |  |  |  |  |  |  |  |  |  |  |  |  |  |  |  |  |  |  |  |  |  |  |  |  |  |  |  |  |  |
| **Weeks** | 22 | 22 | 22 | 22 | 22 | 21 | 21 | 20 | 20 | 21 | 21 | 21 | 20 | 21 | 20 | 21 | 21 | 21 | 20 | 21 | 21 | 20 | 20 | 20 | 20 | 20 | 21 | 20 | 21 | 20 | 20 | 20 | 20 | 21 |
| **Days** | 2 | 5 | 6 | 5 | 4 | 5 | 3 | 6 | 3 | 2 | 1 | 1 | 6 | 0 | 3 | 2 | 0 | 1 | 6 | 0 | 0 | 2 | 0 | 5 | 3 | 4 | 0 | 3 | 2 | 4 | 2 | 0 | 6 | 0 |
| **Ethnicity** |  |  |  |  |  |  |  |  |  |  |  |  |  |  |  |  |  |  |  |  |  |  |  |  |  |  |  |  |  |  |  |  |  |  |
| White | 39 | 68 | 154 | 268 | 298 | 318 | 398 | 520 | 696 | 921 | 1222 | 1623 | 1973 | 2349 | 2435 | 2421 | 2439 | 2218 | 1944 | 1850 | 1860 | 2644 | 2232 | 1802 | 1360 | 1084 | 709 | 595 | 299 | 169 | 81 | 39 | 17 | 6 |
| Other | 2 | 0 | 0 | 0 | 2 | 2 | 5 | 7 | 4 | 9 | 2 | 11 | 15 | 12 | 13 | 9 | 10 | 19 | 8 | 9 | 11 | 17 | 9 | 13 | 7 | 10 | 8 | 4 | 5 | 5 | 2 | 0 | 2 | 0 |
| **Gravidity** |  |  |  |  |  |  |  |  |  |  |  |  |  |  |  |  |  |  |  |  |  |  |  |  |  |  |  |  |  |  |  |  |  |  |
| 1 | 41 | 68 | 154 | 268 | 289 | 303 | 264 | 330 | 449 | 605 | 800 | 1117 | 1321 | 1655 | 1739 | 1745 | 1651 | 1489 | 1123 | 1051 | 1151 | 1370 | 736 | 868 | 642 | 512 | 374 | 267 | 143 | 64 | 35 | 2 | 1 | 0 |
| > 1 | 0 | 0 | 0 | 0 | 12 | 17 | 140 | 197 | 251 | 325 | 424 | 517 | 667 | 706 | 709 | 685 | 798 | 748 | 829 | 808 | 720 | 1291 | 1505 | 947 | 725 | 582 | 428 | 332 | 161 | 110 | 48 | 37 | 18 | 6 |
| **Tabacoo** | 1 | 0 | 1 | 0 | 0 | 3 | 4 | 3 | 1 | 5 | 8 | 8 | 8 | 16 | 9 | 19 | 12 | 14 | 2 | 10 | 25 | 21 | 17 | 12 | 10 | 4 | 2 | 3 | 3 | 1 | 2 | 0 | 0 | 0 |
| **I trimester USG** | **0** | **8** | **28** | **56** | **92** | **151** | **265** | **333** | **527** | **657** | **905** | **1227** | **1477** | **1729** | **1880** | **1919** | **1931** | **1841** | **1516** | **1525** | **1562** | **2524** | **2101** | **1659** | **1236** | **980** | **720** | **452** | **269** | **147** | **68** | **33** | **19** | **6** |
| *% to all pregnancies* | *0,0*  *%* | *11,8%* | *18,2%* | *20,9%* | *30,6%* | *47,2%* | *65,6%* | *63,2%* | *75,3%* | *70,6%* | *73,9%* | *75,1%* | *74,3%* | *73,2%* | *76,8%* | *79,0%* | *78,8%* | *82,3%* | *77,7%* | *82,0%* | *83,5%* | *94,9%* | *93,8%* | *91,4%* | *90,4%* | *89,6%* | *90,2%* | *75,5%* | *88,5%* | *84,5%* | *81,9%* | *84,6%* | *100 %* | *100 %* |
| **Multiple pregnancy** | **1** | **2** | **2** | **1** | **5** | **12** | **11** | **13** | **13** | **32** | **28** | **60** | **78** | **89** | **97** | **106** | **112** | **89** | **75** | **81** | **70** | **89** | **80** | **60** | **42** | **37** | **16** | **17** | **10** | **1** | **1** | **0** | **2** | **0** |
| *% to all pregnancies* | *2,4%* | *2,9%* | *1,3%* | *0,4%* | *1,7%* | *3,8%* | *2,7%* | *2,5%* | *1,9%* | *3,4%* | *2,3%* | *3,7%* | *3,9%* | *3,8%* | *4,0%* | *4,4%* | *4,6%* | *4,0%* | *3,8%* | *4,4%* | *3,7%* | *3,3%* | *3,6%* | *3,3%* | *3,1%* | *3,4%* | *2,0%* | *2,8%* | *3,3%* | *0,6%* | *1,2%* | *0,0%* | *10,5%* | *0,0%* |
| Dichorionic diamniotic | 0 | 2 | 0 | 1 | 2 | 6 | 7 | 6 | 5 | 18 | 15 | 37 | 52 | 52 | 67 | 73 | 68 | 69 | 58 | 60 | 48 | 65 | 64 | 47 | 31 | 30 | 12 | 10 | 5 | 1 | 1 | 0 | 2 | 0 |
| Monochorionic dichorionic | 1 | 0 | 2 | 0 | 2 | 5 | 4 | 6 | 8 | 14 | 11 | 19 | 22 | 34 | 27 | 30 | 35 | 18 | 13 | 18 | 19 | 17 | 13 | 11 | 10 | 7 | 3 | 7 | 4 | 0 | 0 | 0 | 0 | 0 |
| Monochorionic monoamniotic | 0 | 0 | 0 | 0 | 0 | 1 | 0 | 1 | 0 | 0 | 2 | 3 | 0 | 1 | 2 | 1 | 3 | 0 | 1 | 2 | 0 | 1 | 1 | 1 | 0 | 0 | 0 | 0 | 1 | 0 | 0 | 0 | 0 | 0 |
| Dichorionic triamniotic | 0 | 0 | 0 | 0 | 1 | 0 | 0 | 0 | 0 | 0 | 0 | 1 | 0 | 0 | 0 | 1 | 5 | 1 | 1 | 1 | 2 | 4 | 1 | 1 | 1 | 0 | 0 | 0 | 0 | 0 | 0 | 0 | 0 | 0 |
| Monochorionic triamniotic | 0 | 0 | 0 | 0 | 0 | 0 | 0 | 0 | 0 | 0 | 0 | 0 | 1 | 0 | 0 | 0 | 0 | 0 | 0 | 0 | 0 | 0 | 0 | 0 | 0 | 0 | 0 | 0 | 0 | 0 | 0 | 0 | 0 | 0 |
| Trichorionic triamniotic | 0 | 0 | 0 | 0 | 0 | 0 | 0 | 0 | 0 | 0 | 0 | 0 | 1 | 1 | 1 | 1 | 1 | 1 | 2 | 0 | 1 | 2 | 1 | 0 | 0 | 0 | 1 | 0 | 0 | 0 | 0 | 0 | 0 | 0 |
| Trichorionic quadramniotic | 0 | 0 | 0 | 0 | 0 | 0 | 0 | 0 | 0 | 0 | 0 | 0 | 1 | 1 | 0 | 0 | 0 | 0 | 0 | 0 | 0 | 0 | 0 | 0 | 0 | 0 | 0 | 0 | 0 | 0 | 0 | 0 | 0 | 0 |
| Quadrchorionic quadramniotic | 0 | 0 | 0 | 0 | 0 | 0 | 0 | 0 | 0 | 0 | 0 | 0 | 1 | 0 | 0 | 0 | 0 | 0 | 0 | 0 | 0 | 0 | 0 | 0 | 0 | 0 | 0 | 0 | 0 | 0 | 0 | 0 | 0 | 0 |
| **Fetuses (N)** | **42** | **70** | **156** | **269** | **307** | **332** | **415** | **540** | **713** | **962** | **1252** | **1695** | **2072** | **2452** | **2546** | **2538** | **2567** | **2328** | **2030** | **1941** | **1944** | **2756** | **2323** | **1876** | **1410** | **1131** | **815** | **616** | **314** | **175** | **84** | **39** | **21** | **6** |
| **EFW (Grams)** | **417** | **429** | **446** | **465** | **451** | **390** | **365** | **353** | **347** | **370** | **351** | **376** | **352** | **354** | **370** | **360** | **355** | **354** | **368** | **367** | **357** | **381** | **355** | **361** | **376** | **341** | **385** | **348** | **389** | **333** | **303** | **407** | **382** | **352** |
| **Fetal sex** |  |  |  |  |  |  |  |  |  |  |  |  |  |  |  |  |  |  |  |  |  |  |  |  |  |  |  |  |  |  |  |  |  |  |
| Girls | 21 | 36 | 74 | 140 | 147 | 170 | 209 | 259 | 366 | 476 | 619 | 831 | 1041 | 1228 | 1301 | 1282 | 1278 | 1149 | 1026 | 962 | 967 | 1397 | 1141 | 930 | 717 | 567 | 416 | 313 | 152 | 90 | 40 | 20 | 9 | 3 |
| Boys | 21 | 33 | 81 | 129 | 157 | 160 | 206 | 280 | 347 | 486 | 632 | 864 | 1031 | 1223 | 1245 | 1255 | 1288 | 1177 | 1004 | 979 | 977 | 1357 | 1181 | 946 | 693 | 564 | 398 | 302 | 160 | 85 | 44 | 19 | 12 | 2 |
| Undiagnosed | 0 | 1 | 1 | 0 | 3 | 2 | 0 | 1 | 0 | 0 | 1 | 0 | 0 | 1 | 0 | 1 | 1 | 2 | 0 | 0 | 0 | 2 | 1 | 0 | 0 | 0 | 1 | 1 | 2 | 0 | 0 | 0 | 0 | 1 |
| **Invasive procedures** | **0** | **2** | **7** | **16** | **29** | **48** | **32** | **62** | **85** | **119** | **133** | **180** | **233** | **262** | **270** | **293** | **300** | **296** | **293** | **290** | **290** | **470** | **386** | **383** | **351** | **311** | **260** | **196** | **131** | **84** | **35** | **25** | **15** | **5** |
| Amniocentesis | 0 | 2 | 7 | 13 | 27 | 41 | 30 | 58 | 69 | 100 | 116 | 146 | 188 | 219 | 231 | 238 | 254 | 244 | 244 | 248 | 245 | 421 | 355 | 352 | 334 | 282 | 236 | 180 | 122 | 78 | 30 | 23 | 13 | 5 |
| CVS | 0 | 0 | 0 | 3 | 2 | 7 | 2 | 4 | 16 | 19 | 17 | 34 | 45 | 43 | 39 | 55 | 46 | 52 | 49 | 42 | 45 | 49 | 31 | 31 | 17 | 29 | 24 | 16 | 9 | 6 | 5 | 2 | 2 | 0 |
| **Abnormal karyotype** | **0** | **0** | **2** | **4** | **3** | **3** | **2** | **6** | **16** | **18** | **16** | **32** | **39** | **41** | **52** | **55** | **62** | **44** | **43** | **40** | **47** | **60** | **45** | **50** | **41** | **46** | **37** | **39** | **20** | **8** | **7** | **7** | **3** | **1** |
| *% to all fetuses* | *0,0 %* | *0,0 %* | *1,3 %* | *1,5 %* | *1,0 %* | *0,9 %* | *0,5 %* | *1,1 %* | *2,2 %* | *1,9 %* | *1,3 %* | *1,9 %* | *1,9 %* | *1,7 %* | *2,0 %* | *2,2 %* | *2,4 %* | *1,9 %* | *2,1 %* | *2,1 %* | *2,4 %* | *2,2 %* | *1,9 %* | *2,7 %* | *2,9 %* | *4,1 %* | *4,5 %* | *6,3 %* | *6,4 %* | *4,6 %* | *8,3 %* | *17,9%* | *14,3%* | *16,7%* |
| *% to invesive procedures* | *0,0*  *%* | *0,0*  *%* | *28,6%* | *25,0%* | *10,3%* | *6,3%* | *6,3%* | *9,7%* | *18,8%* | *15,1%* | *12,0%* | *17,8%* | *16,7%* | *15,6%* | *19,3%* | *18,8%* | *20,7%* | *14,9%* | *14,7%* | *13,8%* | *16,2%* | *12,8%* | *11,7%* | *13,1%* | *11,7%* | *14,8%* | *14,2%* | *19,9%* | *15,3%* | *9,5 %* | *20,0%* | *28,0%* | *20,0%* | *20,0%* |
